# Checklist for the Structural Description of the Deep Phenotype in Disorders of Sexual Development

**DOI:** 10.1155/2012/816365

**Published:** 2012-02-15

**Authors:** L. Wünsch

**Affiliations:** Department of Paediatric Surgery, University of Lübeck, Ratzeburger Allee 160, 23538, Germany

## Abstract

This paper addresses the question, how the variations of the deep phenotype in disorders of sex development (DSD) are appropriately described. This is a relevant question, because extensive phenotypic variability occurs in gonads and sex ducts. With the advance of video endoscopy and laparoscopy, fresh insight in gonadal and sex duct anatomy is emerging. So far, an attempt to standardize the diagnostic approach and, in particular, how to document these findings has not been published. We propose a standardized examination schedule for these procedures. It consists of 5 pictures of relevant anatomic features. For laparoscopy, it includes two pictures each of gonads and sex ducts on either side and an image of the retrovesical space. For endoscopy, the examination of the ureteric orifices, the posterior urethra, and the urogenital sinus derivates is recommended. Adherence of a standardized schedule and image storing enhances patient autonomy, because they can carry their examination for a second opinion without need for repeated examination. Physicians and scientists create a structured image library that facilitates the comparison of clinical outcomes, research on genotype phenotype associations and may lead to better classifications.

## 1. Introduction

Most disorders of sexual development are characterised by a particular phenotype. Phenotypic variations of the external genitalia have stimulated numerous classification systems. The “deep” phenotype of gonads and sex ducts has not been addressed systematically. Variations of these organs can be detected only by imaging procedures or direct vision. The knowledge of these details is relevant for the clinical management. It is of diagnostic importance when mutational analysis and hormone levels are inconclusive. Gonadal anatomy may also influence the decision of taking a biopsy or performing gonadectomy. Disorders of sex development are rare, making the development of detailed classifications difficult.

Videoendoscopic examination techniques are now widely available. They permit the inspection and imaging of structures that are barely visible with noninvasive imaging procedures. Moreover, images can be stored easily and copied for the patients own purposes. These images may also facilitate the communication among members of the interdisciplinary DSD team. Informed decision making for genital surgery or gonadectomy critically depends on an understanding of how the individual's anatomy deviates from what is defined as “normal.”

Furthermore, there may be a scientific interest in unravelling genotype-phenotype relations. Many new mutations leading to DSD are discovered each year. Relating these new findings to a detailed examination of the “deep” phenotypic variations may foster insight of gene function during urogenital development.

In spite of the highly variable anatomy, affected individuals and their families ask the same questions. Can the gonads be safely retained? Is there a need for surgery and what will be the result?

Gonadectomy and genital surgery are controversial issues, and part of the controversy is about diagnostic uncertainty and lack of standardized procedures. This articles offers a concept for the standardized storage of images taken during endoscopy and laparoscopy.

## 2. Patients and Methods

During 2002 through 2011, 68 patients with DSD underwent diagnostic evaluation at our institution.

Our reports, images, and videos obtained during examinations of various disorders of sex development were reviewed. All patients who had either laparoscopy, endoscopy or both were included.

Cystoscopies and genitoscopies were reexamined with a focus on urethral anatomy, in particular of the posterior urethra.

Laparoscopic images were reviewed with a focus on gonadal and sex duct variations within the same disorder. Diagnosis and procedures are reassumed in Table 1.

## 3. Results

**Figure 1:**
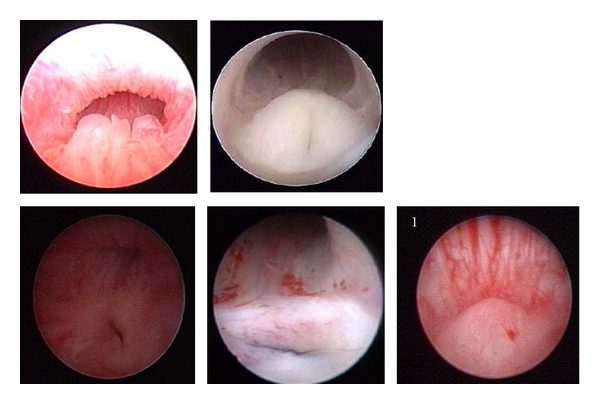
Posterior urethra and utriculus in patients with severe hypospadias and partial gonadal dysgenesis. Size and shape of the colliculus vary from patient to patient. The last image shows a normal colliculus for comparison. Note the different backing of the urethral wall and the difference in shape.

The review of our cystoscopies and genitoscopies showed that a different number of images were stored for each examination. Comparing the images of the posterior urethra in patients with partial gonadal dysgenesis and hypospadias, many variations in the utricular region were found.  Figure 1 shows examples of these variations.

**Figure 2:**
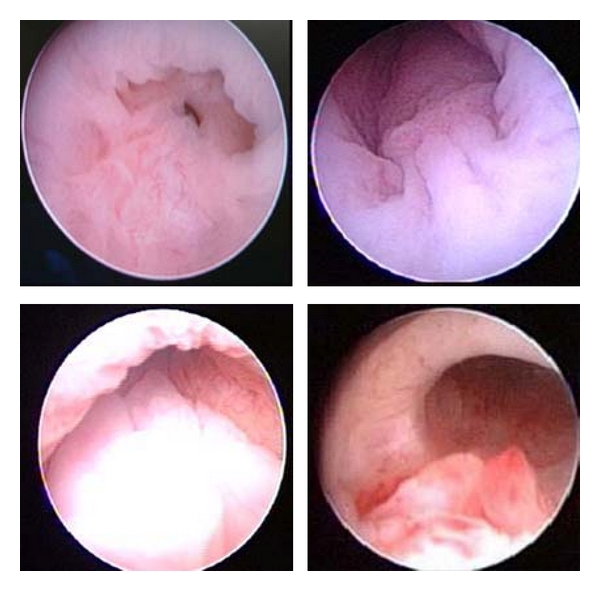
Posterior urethra in congenital adrenal hyperplasia. The area around the vaginal opening is represented. Mucosal folds resemble the hypoplastic verum montanum seen in patients with partial gonadal dysgenesis.

**Figure 3:**
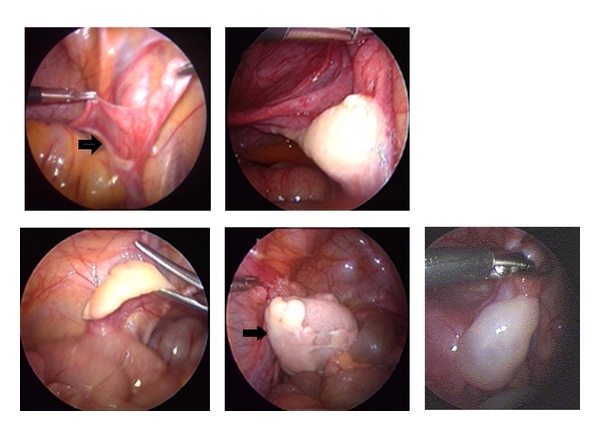
Variations in gonadal shape and size observed in patients with gonadal dysgenesis. The image in the top row on the left shows a streak gonad (arrow). The right lower images show a germ-cell tumour replacing a dysgenetic gonad (arrow). For comparison, a normal postpubertal ovary is shown.

In patients with congenital adrenal hyperplasia, a similar variability was found at the entrance of the vagina to the urogenital sinus (Figure 2). In patients affected by total gonadal dysgenesis, variations of gonadal development are of particular interest. Size and shape of the gonad varied from true streaks to ovoid shape. Examples of this variability and an example of a tumour arising in a gonad are depicted in Figure 3. 

**Figure 4:**
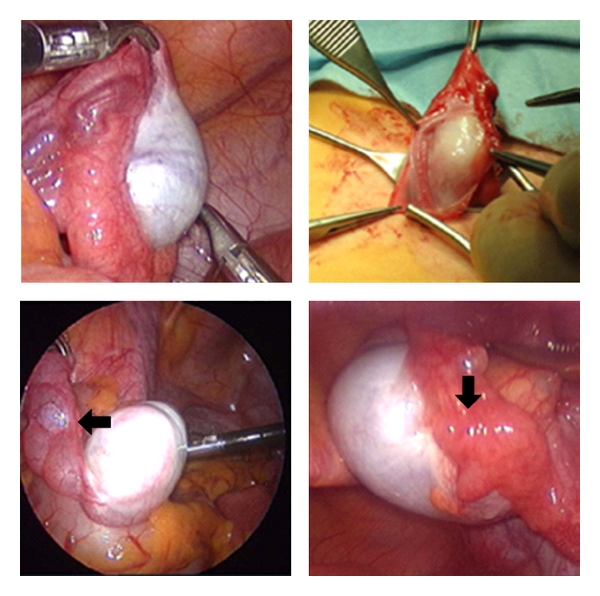
Patients with complete androgene insensitivity have normal appearing gonads in the abdomen or the inguinal canal. Sex duct development is highly variable. The lower panel shows cystic dilatation of an epidydimis, the right lower panel a fallopian tube close to a testis (arrow).

While gonads in androgen insensitivity are very similar to normal testis, a marked variability in their localization and the paramesonephric (Wolffian) structures and even Mullerian structures was evident.  Figure 4 shows examples of this.

**Figure 5:**
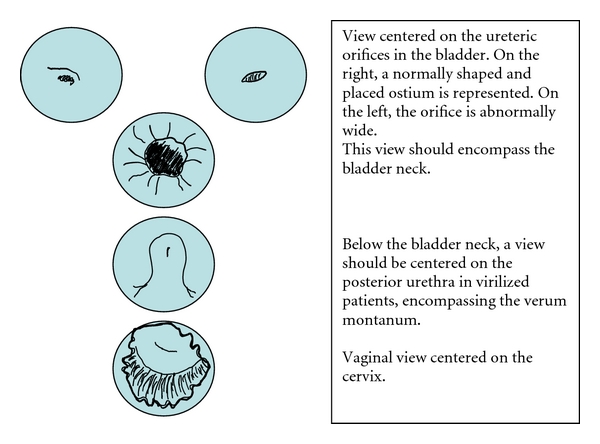
Standard views recommended for cystourethroscopy/genitoscopy. Two images should be taken of the ureteral orifices and one of the bladder neck. Moving downward, the bladder neck, the posterior urethra with the colliculus and the vagina/cervix should be documented.

**Figure 6:**
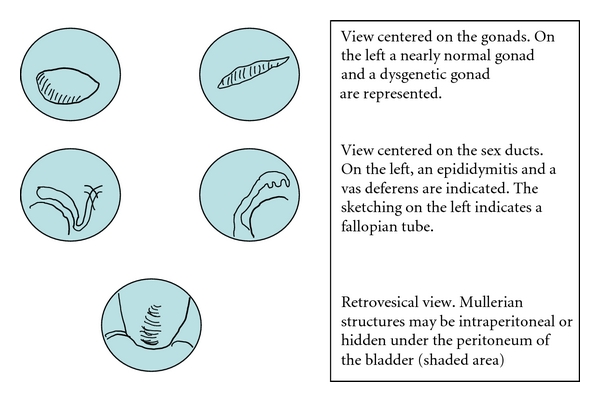
At laparoscopy, 2 images should be taken of each gonad and the attached sex duct. The retrovesical space should be explored for the presence an uterus or vas deferens.

These examples show convincingly that very different “deep phenotypes” may exist within a given disorder. These observations prompted us to recommend a standardized examination schedule with a precise proposal on what images to take. Photographic views are referred to defined anatomic landmarks as shown in Figures 5 and 6.

## 4. Discussion

Our own experience and a review of the literature showed a wide variability of diagnostic procedures and terminology that was used to describe the deep phenotype in DSD. We focussed on endoscopy and laparoscopy, because compared to the noninvasive investigations they allow the best visualisation. However, techniques and reporting of findings are also subject to variation and we concluded that a standardization of the surgical evaluation and defining what images to store would be most useful. Based on the reevaluation of 66 patients with various DSD treated at our institution we propose the examination schedule shown above. All the views are clearly defined, refer to stable anatomic landmarks, and are easily applicable.

Storing of images is helpful for the interested individual and those who offer counselling or care. DSD requires a holistic approach, and the communication between team members is likely to be more efficient, when relevant anatomic details can be shared easily [1]. Both patients and doctors can obtain second opinions for rare or controversial clinical situations.

Documenting anatomical findings in detail and high quality may improve future outcome studies. Size, shape, and surface anatomy of the gonads are highly variable, and these factors may be relevant for the individual tumour risk. The endoscopic findings are important for the planning of genital reconstruction. The distance between bladder neck and vaginal confluens is important for vaginal reconstruction and continence [2]. Anomalies of the verum montanum are common and contribute to the influx of urine. The vaginal dimensions and relations to urethra are important for sexual function. Several noninvasive imaging procedures have been described [3], but the quality of endoscopy and laparoscopy for the visualisation of small mobile structures is unparalled [4].

For most rare conditions, outcome research is badly needed but difficult to obtain. Images taken in a standardized fashion can be pooled to create bigger patient populations. They can be analyzed applying checklists, and interobserver bias and observer bias can be addressed. Phenotypic features can be also evaluated retrospectively. This may facilitate clinical decision making, create a comparable data base for future investigations, and improve quality by eliminating ambivalent terminology. Furthermore, the definition of the normal phenotype is in continuous evolution [5]. Endoscopy and imaging techniques not only delineate pathologic findings, but also contribute a comprehensive definition of the normal phenotype and its variations.

## Figures and Tables

**Table 1:** Patients, diagnosis, and procedures.

Diagnosis	No. of patients	Procedures
Complete androgen insensitivity	8	Genitoscopy, laparoscopy, herniorrhaphy
Complete gonadal dysgenesis	12	Genitoscopy, laparoscopy, gonadectomy
Partial gonadal dysgenesis	12	Genitoscopy, laparoscopy, genitoplasty
Defects of androgen synthesis	6	Genitoscopy, laparoscopy, genitoplasty
Ovotesticular DSD	3	Genitoscopy, laparoscopy
Congenital adrenal hyperplasia	17	Genitoscopy
Turner syndrome	3	Genitoscopy, laparoscopy
Partial androgen insensitivity	1	Genitoscopy, laparoscopy
Frazier syndrome	1	Genitoscopy, laparoscopy
Unclear diagnosis	5	Genitoscopy, laparoscopy
